# Have we got the optimal treatment for refractory Kawasaki disease in very young infants? A case report and literature review

**DOI:** 10.3389/fped.2023.1210940

**Published:** 2023-07-28

**Authors:** Robert Lersch, Guido Mandilaras, Meike Schrader, Felicitas Anselmino, Nikolaus A. Haas, André Jakob

**Affiliations:** ^1^ Department of Paediatric Cardiology and Paediatric Intensive Care, Ludwig-Maximillian-University Munich, Munich, Germany; ^2^ Department of Paediatrics, Clinic Starnberg, Starnberg, Germany

**Keywords:** Kawasaki disease, tumor-necrosis-factor-alpha inhibitor, coronary artery aneurysm, young infant, inflammation

## Abstract

A small group of patients with Kawasaki disease (KD) demonstrates resistance to standard therapy, putting them at high risk for an unfavorable prognosis, especially regarding coronary artery aneurysms. Although adding corticosteroids to first-line i.v. immunoglobulins (IVIGs) is considered beneficial, and despite timely treatment initiation, very young infants, in particular, can present an unfavorable clinical course. We report on a 3-month-old boy with a clinically severe KD phenotype involving the early development of giant coronary artery aneurysms. Because of his poor response to the first course of IVIG and prednisolone, we administered infliximab. His clinical condition improved after that, and his temperature dropped. Inflammatory markers however did not recover completely, and he remained subfebrile. In addition, as the coronary artery dimensions deteriorated, a second IVIG course was administered and prednisolone continued at the initial dosage. Although fever and routine inflammatory parameters normalized, close follow-up investigations revealed both still increasing coronary artery dimensions and renewed rise in inflammatory parameters, necessitating two more infliximab administrations in addition to continuous prednisolone. Because of the coronary artery dimensions (left anterior descending artery, 4.9 mm, Z-score 11.1; right coronary artery 5.8 mm, Z-score 15.5), dual platelet inhibitory therapy with ASA and later clopidogrel combined with low-molecular heparin was indicated. Four weeks after his initial KD diagnosis, we detected no renewed increase in inflammatory markers; at that time, we observed a slight reduction in coronary dimensions. In summary, despite timely guideline-fulfilling therapy, the prolonged clinical course of this very young infant with KD entailing the development of giant coronary artery aneurysms makes us question whether this age group may benefit from early, even more intense therapy.

## Introduction

Kawasaki disease is a disease that causes acute systemic vasculitis in children, which results in coronary aneurysms in up to 25% of patients (1). The classical symptoms include high fever, conjunctival injections, fissured lips, oral mucosa anomalies, erythema and edema of the extremities, cervical lymphadenopathy, and diffuse exanthema (2). The worst complication of KD, especially in terms of mortality and morbidity, is coronary artery aneurysms (CAAs) (3). Standard treatment includes high doses of intravenous immunoglobulins (IVIGs) and acetylsalicylic acid (ASA) (4). Only IVIGs have significantly reduced CAAs so far, with good tolerability, when administered early (5, 6). In the hope of lowering CAA rates even further in KD children, corticosteroids are being increasingly applied as additional first-line treatment. Following the available data on first-line treatment, corticosteroids are recommended in those KD children who expose a risk predisposition of being refractory to IVIG and thus CAA development (7). Several factors are known to significantly raise the risk of developing CAAs, i.e., young age is considered relevant, particularly in infants under 1 year of age at disease onset (8–10). In nonresponsive patients with persistent fever, second-line therapy includes a second course of IVIG or continued therapy with biologicals such as anakinra or infliximab, high-dose corticosteroids, or cyclosporine A (1, 11–14). According to a recent randomized trial and meta-analysis, infliximab may be superior to a second IVIG infusion with good tolerability (15, 16).

We report on a severely affected 3-month-old child with KD unresponsive to the first IVIG infusion and prednisolone. Close follow-up monitoring revealed renewed inflammatory processes treated with infliximab, followed by a second IVIG infusion and concomitant prednisolone. However, despite this timely and intensive treatment regimen, his CAA dimensions developed into giant aneurysms within the first weeks of the disease.

## Case report

A 3-month-old boy presented with a high fever (40 °C), nasal congestion, coughing, and reduced general condition at a regional hospital. Physical examination initially showed a reddened throat and prolonged capillary refill time with no evidence of rash or lymphadenopathy. Laboratory examination revealed elevated C-reactive protein (CrP, 9.6 mg/dl). A bacterial infection was suspected, and antibiotic therapy with ampicillin/sulbactam was initiated. Given the worsening clinical condition, increasing infection parameters, and ongoing suspicion of a bacterial infection without any detected pathogen thus far, a cerebrospinal fluid puncture was performed. With mild pleocytosis in the cerebrospinal fluid, the antibiotic treatment was then escalated to ceftriaxone. Shortly after that, 5 days after fever initiation, the patient developed a pale pink, subtly spotted maculopapular rash particularly evident on the upper thorax, palms, and soles and a bilateral conjunctivitis without exudation and fissured lips. Prominent lymphadenopathy was observed, characterized by lymph nodes with a diameter of up to 2 cm, primarily affecting the cervical region and the nuchal, retroauricular, and inguinal areas. He also passed green, liquid stools.

Additional family history: At the age of 2 weeks, the boy was hospitalized with a protracted upper-airway viral infection. The mother had had a COVID-19 infection during late pregnancy; otherwise, her pregnancy course was without complications. The father had experienced an undetermined inflammatory event triggering autoimmune hemolytic anemia at age 20 years. Both parents are of German descent.

Clinical course: Initially, the patient was referred to our department due to the worsening of his general condition and increased CrP levels. Laboratory evaluation revealed elevated CRP (19.1 mg/dl), hypoalbuminemia (2,4 g/dl), normocytic anemia (Hb 8,6 g/dl), mild leukopenia (5,2 G/L), and mild thrombocytopenia (123 G/L). Virological screening, including infection with adenovirus, enterovirus, HSV, HHV6, and hepatitis, was negative. Echocardiography showed a right coronary artery ectasia [RCA, right coronary artery: Z-score 2.3 according to Dallaire and Dahdah (17)] and mild pericardial effusion, while abdominal sonography revealed mild free peritoneal fluid. Considering the patient's clinical presentation, including low values of white blood cells and platelets, macrophage activation syndrome (MAS) was considered a potential differential diagnosis and possibly being triggered by KD. However, based on subsequent investigations, such as normal levels of ferritin and triglycerides and the absence of splenomegaly on ultrasound and coagulopathy, MAS was rather unlikely.

The patient developed periorbital and extremity edema, and the final diagnosis of complete KD was made. Treatment with IVIG (2 g/kg) and high-dose ASA (30 mg/kg/day) was initiated, along with prednisolone (2 mg/kg/day) due to additional risk factors (age under 1 year, initially enlarged coronary arteries) (7). Despite the first course of IVIG, the patient's fever persisted longer than 36 h, the general condition did not improve significantly, and inflammatory parameters showed only minimal reduction. The coronary artery dimensions continued to expand (Z-score: RCA: 3; LAD, left anterior descending artery: 4.5). Accordingly, second-line therapy with infliximab (10 mg/kg) was initiated following the latest recommendations (15). However, the patient remained subfebrile after infliximab, and inflammatory markers indicated persistent inflammation with further enlargement of the coronary arteries (Z-score: RCA: 7.8; LAD: 5). A second IVIG course was administered, finally leading to a resolution of fever and normalization of inflammatory parameters. If additional escalation steps were deemed necessary, we would have considered using steroid boluses or alternative biological agents, such as anakinra.

**Figure 1:**
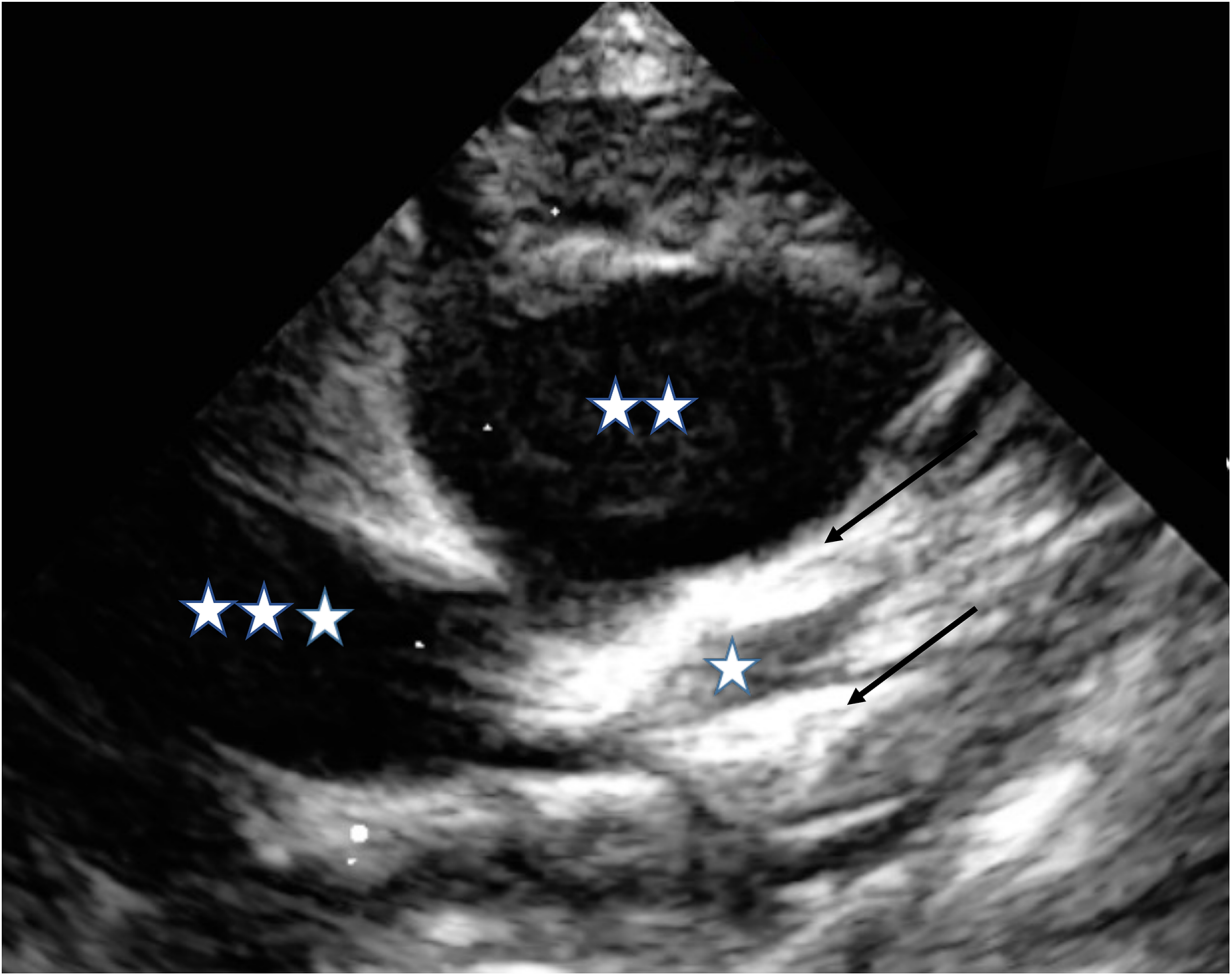
“Bright” appearing perivascular tissue in the echocardiography around CAA. *LAD, left coronary artery; **PA, pulmonary artery; ***AO, aorta; arrow; “bright” appearing perivascular tissue.

Following the second IVIG administration, the coronary artery aneurysms continued to enlarge, prompting the continuation of corticosteroid treatment and the initiation of low-molecular-weight heparin. Despite the further increase in coronary artery dimensions, inflammatory markers did not increase, with no pathologically activated T lymphocytes (CD3+DR+) and normal von Willebrand and factor VIII levels. We therefore decided against expanding the immune modulatory therapy further (anakinra vs. cyclosporin). Close outpatient follow-up was emphasized, and the patient experienced a further increase in coronary artery dimensions 1 week later. Additionally, we observed a slight elevation in inflammatory parameters (CRP 2.1 mg/dl, leukocytes 23 G/I) and a positive troponin T (0.078 ng/ml). However, the electrocardiography results did not indicate severe coronary ischemia, regional myocardial dyskinesia, or the presence of a visible coronary artery thrombus within the examined segments. To address the possibility of reactivation of Kawasaki disease-related inflammation, we opted to initiate a second infusion of infliximab (10 mg/kg/day) and introduce dual-platelet inhibition with clopidogrel alongside ASA in addition to the ongoing administration of low-molecular-weight heparin. Despite a rapid normalization of inflammatory parameters, a third infusion of infliximab (5 mg/kg/day) was administered 1 week later due to the development of giant coronary artery dimensions (LAD: 4.9 mm, Z-score: 11.1; RCA: 5.8 mm, Z-score: 15.5) and persistent perivascular brightness (Figure 1). Throughout the patient's clinical course, there were no indications of shock or the need for intensive care unit hospitalization. During subsequent follow-up visits, we observed an improvement in coronary artery dimensions (e.g., 4 months after initial diagnosis: LAD 2.3 mm, Z-score 3.5; RCA 4.8 mm, Z-score 10) and found no signs of inflammation. Gradual tapering of corticosteroid treatment was implemented and completed 80 days after initiation. Given the presence of persistent giant aneurysms and elevated troponin T levels, the patient continues to receive anticoagulation and dual-platelet inhibition until coronary angiography is conducted at least 6 months after the acute phase of the disease.

**Figure 2:**
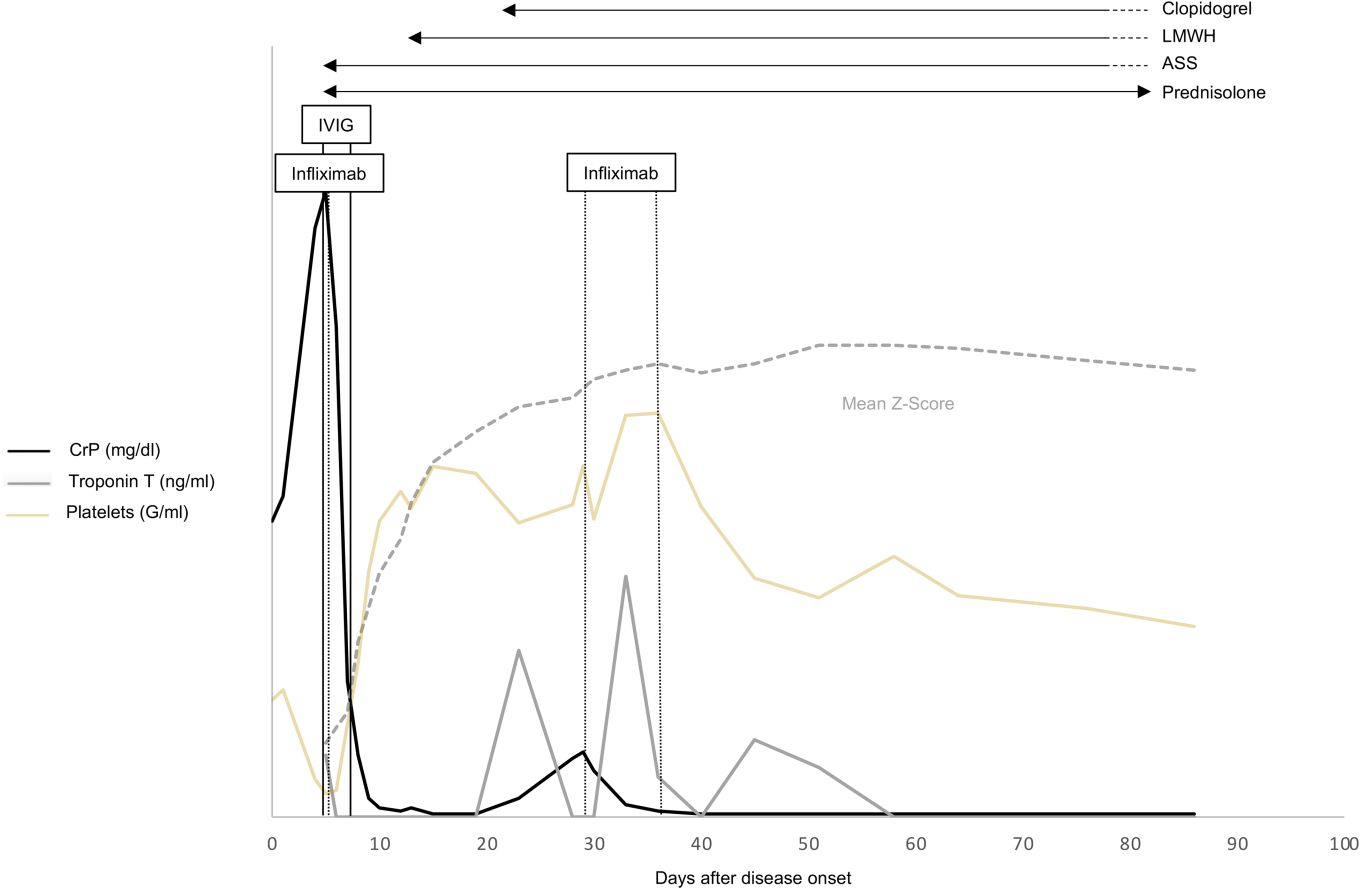
Time course of clinical Kawasaki disease (KD) signs and laboratory values in relation to the prescribed KD medications (ASA, prednisone, IVIG, infliximab) and days after disease onset. LMWH, low-molecular-weight heparin; ASA, acetylsalicylic acid; IVIG, immunoglobulin; CrP, C-reactive protein.

Figure 2 provides a comprehensive overview of the therapy timeline, the progression of Z-scores in coronary arteries, and the key laboratory parameters.

## Discussion

We report on a very young patient presenting the phenotype of severe KD who developed giant coronary artery aneurysms despite timely treatment following respective guidelines and the latest evidence.

Young infants represent a special KD subgroup suffering more frequently from incomplete clinical presentation, eventually leading to later correct diagnosis and treatment initiation (Table 1). Resistance to IVIG and pronounced involvement of the coronary arteries are more common in this age group (18, 19), but this is only partially attributable to later treatment initiation. In our case, 2 g/kg of IVIG, ASA 30 mg/kg/day was directly combined with 2 mg/kg/day of prednisolone, as this boy was considered a “high-risk” case. The Japanese risk scores used in this context do not reliably reveal “high risk” among Caucasian children (20). Nevertheless, as several individual disease-related aspects, such as young age, are associated with IVIG resistance, adding corticosteroids to first-line IVIG treatment is therefore recommended as a therapeutic option among several national guidelines (11). However, we failed to observe any response to this therapy in our case. Resistance to immunoglobulins is defined as fever persisting longer than 36 h after administration (21). A second IVIG infusion combined with steroids is usually recommended in this situation (11). However, additional therapeutic options such as TNF alpha inhibitors, interleukin-1 receptor antagonists, and cyclosporin can also be applied. Recent data on Caucasian children suggest that early infliximab therapy may have advantages over a second cycle of IVIG (15, 16) (Table 1). However, it was only after the second IVIG administration that this child improved clinically and his subfebrile temperature and infection parameters normalized (see Figure 2).

**Table 1:** Summary of the literature on refractory KD in very young infants and the use of infliximab and anakinra in refractory disease.

Publication	Major findings	Study design	Cohort size	PMID
Resistance to standard treatment in very young infants				
Kobayashi et al. (2006)	Higher risk of developing resistance to IVIG in younger patients, patients with low sodium and platelet count, and patients with high levels of CrP and AST	Retrospective	546	16735679
Mastrangelo et al. (2019)	Very young infants are more likely to have incomplete KD, more likely to show coronary artery involvement and higher resistance to IVIG treatment	Retrospective	113	31493782
Egami et al. (2006)	Younger age, longer illness duration, low platelet count, and high ALT and CrP levels are associated with IVIG-unresponsiveness	Randomized controlled multicenter trial	320	16887442
Rosenfeld et al. (1995)	Infants younger than 1 year are at greater risk of developing giant CAA	Retrospective	443	7699529
Kim et al. (2023)	Age under 1 year and resistance to the first IVIG treatment are associated with a higher recurrence rate	Retrospective	19,456	36549869
Garcia et al. (2020)	KD in patients under 3 months of age is more often incomplete and shows a higher risk of cardiac complications	Retrospective	688	32793527
Studies on infliximab for the treatment of KD				
Burns et al. (2021)	Infliximab might lead to a shorter duration of fever and shorter hospitalization stay for patients with IVIG-resistant KD compared to a second IVIG infusion	Randomized controlled multicenter trial	105	34715057
Masuda et al. (2017)	Infliximab seems to be effective in treating refractory KD in a cohort of 434 patients	Retrospective	434	29224935
Hur et al. (2019)	Early use of infliximab might reduce the incidence of pronounced CAA in patients with refractory KD	Retrospective	102	30468032
Son et al. (2020)	Infliximab therapy was associated with a shorter fever duration and shorter hospitalization stay	Retrospective	106	21129756
Miyata et al. (2023)	Initial adjunctive therapy with infliximab is associated with a higher CAA regression rate in patients with initial CAAs	Retrospective	168	37258054
Tremoulet et al. (2014)	Addition of infliximab to initial therapy reduced fever duration, inflammation markers, and left anterior descending coronary artery Z-score in KD patients	Randomized controlled phase III trial	196	24572997
Mori et al. (2018)	Higher rate of defervescence in KD patients with initial IVIG resistance receiving infliximab compared to a second IVIG infusion	Randomized controlled phase III trial	31	29386515
Nagatomo et al. (2018)	Infliximab therapy may be effective for the early improvement of CAAs in KD patients with IVIG resistance	Retrospective	49	30144998
Li et al. (2021)	Infliximab is effective for patients with IVIG resistance and leads to a shorter and milder clinical course	Meta-analysis		33652059
Studies on anakinra for the treatment of KD				
Yang et al. (2021)	Anakinra therapy is safe in patients with KD	Randomized controlled phase I/IIa trial	22	34953816
Koné-Paut et al. (2020)	Anakinra may reduce fever duration, inflammation markers, and coronary artery dilation in patients with IVIG-refractory KD	Phase II open-label study	16	32779863
Koné-Paut et al. (2018)	Anakinra used in the late disease course led to reduced inflammation	Retrospective	11	29885546

We can only speculate whether this infant would have responded better to an earlier second IVIG infusion or whether the development of giant CAAs could have been prevented by earlier, even more intense treatment. We recently reported on a similarly very young KD infant who developed a giant coronary artery aneurysm during the acute disease course. That child's inflammation was only stopped by a combination of biologicals (first anakinra, then combined with etanercept), which indeed we had only applied after the infant continued to be refractive to two courses of IVIG and corticosteroids (22).

There is study evidence that young infants, i.e., less than 1 year of age, can suffer from severe clinically progressing KD (10, 19) and are resistant to standard therapy, with a high relapse rate identified as a major contributing factor. A recent nationwide cohort study conducted in Korea with nearly 20,000 patients again reported a significantly higher recurrence rate in patients younger than 1 year of age (23). This raises the question of whether acute-phase treatment should be modified differently for these very young KD patients.

In terms of outpatient follow-up, according to American Heart Association and German guidelines, KD patients presenting evolving coronary artery abnormalities (Z-score > 2.5) should be echocardiographically monitored at least twice per week (1). In our case, we mandated close clinical follow-up visits combining echocardiographic and electrocardiographic evaluation with blood sampling. Although the child remained clinically afebrile and unimpaired, we observed a renewed increase in inflammatory parameters and perivascular brightness, such as a moderate increase in troponin T. Nevertheless, it is important to note that CrP levels may not necessarily reflect inflammation with ongoing corticosteroid treatment, emphasizing the need for close combined clinical, echocardiographic, and laboratory follow-up.

In our case, it was only after we had applied three cycles of infliximab and prolonged therapy with high-dose steroids combined with anticoagulation and dual anti-platelet therapy that we stopped detecting recurring inflammation in our patient, as well as no troponin T and regressing coronary artery dimension.

The distinctiveness of our case includes the very young age of the patient, the rapid progression of coronary artery aneurysms despite early treatment initiation on day 5 of fever, and the presence of renewed inflammation during the follow-up period under prednisolone treatment. Notably, it was only after administering three cycles of infliximab and implementing prolonged therapy with high-dose steroids in combination with anticoagulation and dual antiplatelet therapy that we observed cessation of recurring inflammation, as well as a decline in troponin T levels and regression of coronary artery dimensions.

## Conclusion

This case report illustrates the severe clinical course of a 3-month-old infant with KD who developed giant coronary artery aneurysms despite early guideline-based therapy. Furthermore, by ensuring thorough follow-up evaluations, we were able to detect recurring inflammation and indications of a possible coronary blood-flow disorder.

Young infants are known to respond poorly to KD-specific therapy and to suffer from severe disease progression. They are also more likely to present with recurrent inflammation. However, neither one specific treatment nor follow-up evaluation is recommended for this age group. We believe that this delicate age group deserves more intense research efforts and discussion, as these children may benefit from a specific treatment recommendation in the future.

## Data Availability

The original contributions presented in the study are included in the article, further inquiries can be directed to the corresponding author.
